# Microbiological and Physicochemical Characterization of Irrigation Water in Market Gardening in Korhogo

**DOI:** 10.1155/2022/3167581

**Published:** 2022-05-25

**Authors:** Ollo Kambire, Konan Mathurin Yao, Natchia Aka, Karidja Emilie Bamba, Rose Koffi-Nevry

**Affiliations:** ^1^Department of Biochemistry and Genetics, Peleforo Gon Coulibaly University, BP 1328, Korhogo, Côte d'Ivoire; ^2^Oceanographic Research Center, BP V 18, Abidjan, Côte d'Ivoire; ^3^Laboratory of Biotechnology and Food Microbiology, Department of Food Science and Technology, Nangui Abrogoua University, 02 BP 801, Abidjan, Côte d'Ivoire

## Abstract

Several sources of water are used by farmers without concern for quality, which can have consequences on the health of the consumer of market garden products. The aim of this study is to evaluate the microbiological and physicochemical qualities of irrigation water. Microorganisms were counted using the membrane filtration and incorporation into the agar methods. The physicochemical parameters were measured using multiparameter and spectrophotometric methods. The average values of the physicochemical parameters are between 6.46 and 6.9 (pH), 27.15 and 29.9°C (temperature), 170 and 760 *μ*S/cm (electrical conductivity), 70 and 380 mg/L (total dissolved solids), 3.85 and 77.59 mg/L (nitrates), and between 0.13 and 2.35 mg/L for ammonium. Irrigation water in market gardening is highly contaminated by microorganisms. Loads ranging from 3.64 to 4.35 log_10_ cfu/100 mL, 2.44 to 3.31 log_10_ cfu/100 mL, 2.44 to 2.9 log_10_ cfu/100 mL, and 2.07 to 3.63 log_10_ cfu/100 mL were obtained for total coliforms, *E. coli*, fecal enterococci, and sulphite-reducing clostridia, respectively. Mean loadings ranging from 4.95 to 5.98 log_10_ cfu/100 mL, 1.8 to 2.08 log_10_ cfu/100 mL, and 1.5 to 1.98 log_10_ cfu/100 mL were obtained for mesophilic aerobic germs, moulds, and yeasts, respectively. Four different mould strains were identified in irrigation water. These strains belong to the genus *Aspergillus*. Shallows water was more contaminated with microorganisms. These results show that water should be treated before being used for irrigation; market garden products must be properly washed and disinfected before consumption.

## 1. Introduction

The world urban population is at an all-time high, and the share of urban dwellers is projected to represent two-thirds of the global population in 2050 [1]. By 2025, more than two-thirds of the world's population will live in cities [2]. Thus, for many years, governments in Francophone Africa have been faced with the phenomenon of migration to urban centers. In addition to demographic implications (unemployment and impoverishment of a majority of urban populations), this migratory flow has had negative repercussions on the environment, available resources, and food [3]. Population growth by 2050 will lead to a high food demand to ensure food security [4]. In this context, to meet consumer demands, urban and periurban agriculture through its specialization in fruit and vegetable production is now considered a viable and sustainable solution to counter food insecurity [2]. Urban agriculture has attracted the attention of academics, policy-makers, and practitioners alike as a potential measure to address the food needs of growing city populations and counter some of the negative environmental and economic effects of urbanization [5]. Currently, one of the most important challenges to achieve food security is the intensification of global food production. Most surveys and research efforts in agriculture focus on crop production [6].

In Côte d'Ivoire, market gardening appears to be one of the main agricultural activities for supplying fruit and vegetables in the cities. Since the 1990s, dry season production of fresh vegetables for cities in Côte d'Ivoire has spread to the Senufo savannahs on the Ivorian-Burkinabe border [7]. Market gardening is one of the main sources of income for most communities in northern Côte d'Ivoire. It is practiced at any time of the year, in the rainy season as in the dry season, generally around streams, reservoirs, and wells [8]. Market gardening is practiced in Korhogo, a city located in the north of Côte d'Ivoire, where the water deficit is chronic. Thus, the water resources at its disposal are faced with problems of availability, quality, and integrated and sustainable management. Indeed, these water resources suffer among other things from problems related to population growth in recent years and high vulnerability to climate hazards [9].

Market gardening is dependent on the availability of water. The water used for irrigation in market gardening is most often polluted. Thus, according to Amoah et al. [10], in sub-Saharan Africa, 10% of the urban population uses wastewater for crop irrigation, with 50–90% of urban dwellers in West Africa having consumed vegetables irrigated with wastewater or polluted surface water. In Korhogo, the irrigation water is made up of water from dams, wells, and shallows. These water points are not protected and can be contaminated by organic and microbial elements. The use of this water for watering could lead to strong contamination of vegetables by microorganisms, some of which could be dangerous for the consumer. According to Koffi-Nevry et al. [11], cases of food poisoning related to ingestion of contaminated vegetables have been identified around the world. Among the factors commonly implicated in vegetable contamination is irrigation water [12].

The aim of this study is to evaluate the microbiological and physicochemical qualities of irrigation water in order to prevent cases of food poisoning related to consumption of market garden products.

## 2. Materials and Methods

### 2.1. Study Area

Located between 10°41′ and 8°53′ north latitude and 5°30′ and 6°31′ west longitude and 635 km from Abidjan, the city of Korhogo is the administrative center of the Savanes district and the Poro region. It is the fourth largest city in Côte d'Ivoire, in terms of population and economy. As a department, it covers an area of 12,500 km^2^ or 3.9% of the national territory.

### 2.2. Sampling

**Figure 1:**
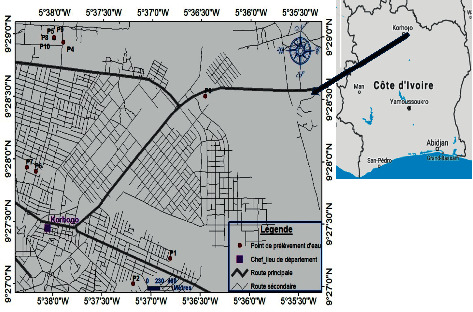
Sampling sites.

The sampling was carried out during the dry season. Ten sites were selected in the market gardening area of the city of Korhogo for the collection of water samples (Figure 1). At each site, two water samples were collected using the farmers' seal, one sample for microbiological analysis and the second for physicochemical analysis. Water samples were taken from shallows (2 sites), dam (4 sites), and well (4 sites). All samples collected were transported to the laboratory in a cooler containing ice.

### 2.3. Physicochemical Analysis

The physicochemical parameters (pH, temperature, total dissolved solids, and electrical conductivity) were measured in situ using a multiparameter.The multiparameter electrode was immersed directly in water for simultaneous reading of the four parameters. Ammonium and nitrate were determined using spectrophotometry. In alkaline medium and in the presence of nitroprusside which acts as a catalyst, ammonium ions treated with a solution of chlorine and phenol give indophenol blue which can be determined at wavelength of 630 nm. Nitrates give yellow colored sodium paranitrosalicylate, which can be determined at wavelength 415 nm in the presence of sodium salicylate.

### 2.4. Microbiological Analysis

#### 2.4.1. Enumeration of Microorganisms

The quantification of aerobic mesophilic germs and sulphite-reducing anaerobic bacteria was performed by the pour plate method proposed by Rodier et al. [13]. After the destruction of vegetative forms, spores of sulphite-reducing clostridia were enumerated on TSN agar (BioRad, France). Mesophilic aerobic germs were enumerated on plate count agar (Merck, Germany). The quantification of total coliforms, *Escherichia coli*, fecal enterococci, sulphite-reducing clostridia, yeasts, and moulds were carried out using the filtration technique [13]. All water samples were diluted prior to filtration on 0.45 *μ*m diameter cellulose ester membranes. Total coliforms and *E. coli* were enumerated simultaneously on RAPID'*E. coli* 2 agar (BioRad, France). Confirmation of the presence of *E. coli* was carried out by the indole urea test. The media Slanetz and Bartley (Oxoid, UK), Dichloran Rose Bengal Chloramphenicol (Oxoid, UK), and TSN (BioRad, France) were used for the enumeration of fecal enterococci, yeasts, moulds, and the spores of sulphite-reducing clostridia, respectively. All inoculated Petri dishes were incubated at 37°C.

#### 2.4.2. Mould Identification

After the purification of the mould strains, their identification was carried out based on macroscopic and microscopic characters from the identification keys published by David Malloch translated and adapted by Lecomte [14].

### 2.5. Statistical Analysis

The calculation of means and standard deviations was performed with XLSTAT 2020 software. Coinertia using the ade4TkGUI software was used to establish correlations between physicochemical and microbiological parameters.

## 3. Results

### 3.1. Microbiological Analysis

#### 3.1.1. Total Coliforms

**Figure 2:**
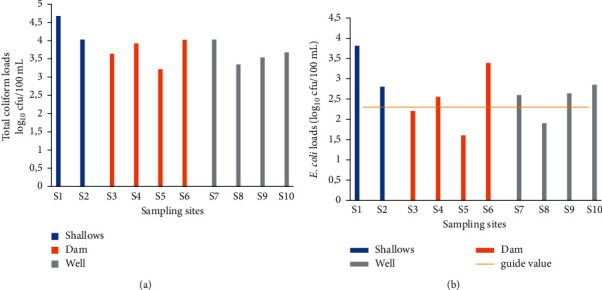
Total coliform (a) and *E. coli* (b) counts in irrigation water.

The total coliform count in irrigation water (Figure 2(a)) ranged from 3.21 to 4.67 log_10_ cfu/100 mL. The highest load (4.67 log_10_ cfu/100 mL) was obtained in site 1 and the minimum load (3.21 log_10_ cfu/100 mL) in site 5.The highest average load (4.35 log_10_ cfu/100 mL)was obtained in shallow water, followed by dam water (3.7 log_10_ cfu/100 mL) and well water (3.64 log_10_ cfu/100 mL) (Table 1).

#### 3.1.2. *Escherichia coli*

*E. coli* counts in irrigation water ranged between 1.6 and 3.81 log_10_ cfu/100 mL (Figure 2(b)). The maximum load (3.81 log_10_ cfu/100 mL) and minimum load (1.6 log_10_ cfu/100 mL) were obtained at sites 1 and 5, respectively. *E. coli* counts of dam (sites 3 and 5) and well (site 8) water were below the guide value (2.3 log_10_ cfu/100 mL). All shallow water loads were above the guide value. Mean loads in irrigation water were 3.31, 2.44, and 2.5 (3.81 log_10_ cfu/100 mL) for shallows, well, and dam water, respectively (Table 1). The shallow water was the most contaminated with *E. coli*.

#### 3.1.3. Fecal Enterococci

**Figure 3:**
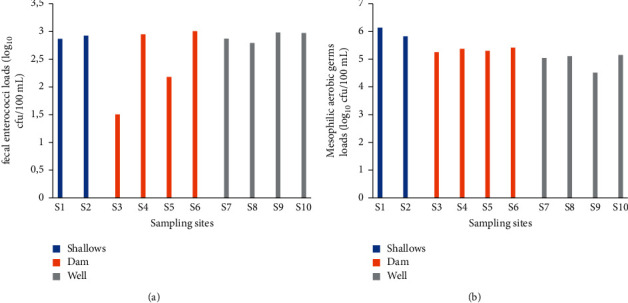
Fecal enterococci (a) and mesophilic aerobic germs (b) counts in irrigation water.

The maximum (3 log_10_ cfu/100 mL) and minimum (1.5 log_10_ cfu/100 mL) fecal enterococci loads were obtained at sites 6 and 3, respectively (Figure 3(a)). Apart from the load at site 3, the other recorded loads were of the same order. The average fecal enterococcal loads (Table 1) obtained were 2.89, 2.44, and 2.9 log_10_ cfu/100 mL for shallow, dam, and well water, respectively. Well and shallow water were the most contaminated with fecal enterococci.

#### 3.1.4. Mesophilic Aerobic Germs

Mesophilic aerobic germ count ranged from 4.51 to 6.13 log_10_ cfu/100 mL (Figure 3(b)). These loads were obtained in sites 9 and 1, respectively. Apart from the loads from these two sites, the loads obtained in irrigation water from the other sites were of the same order. The average loads obtained in shallow, dam, and well irrigation water (Table 1) were 5.98, 5.34, and 4.95 log_10_ cfu/100 mL, respectively. Water from shallows was the most contaminated followed by water from the dam and well.

#### 3.1.5. Sulphite-Reducing Clostridia

**Figure 4:**
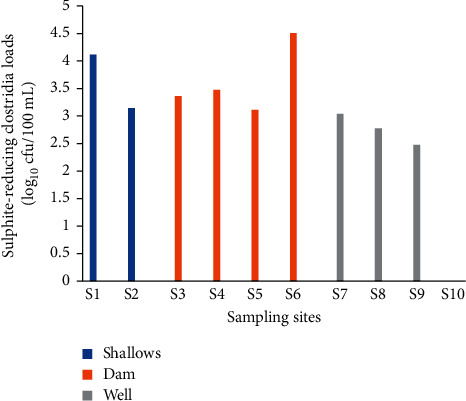
Sulphite-reducing clostridia counts in irrigation water.

Loads ranging from 0 to 4.5 log_10_ cfu/100 mL of spore were obtained in irrigation water (Figure 4). Site 6 was the most contaminated with sulphite-reducing clostridia spores. No spores were obtained from the water of site 10. The highest average load (3.63 log_10_ cfu/100 mL) was obtained in shallow water, followed by dam water (3.61 log_10_ cfu/100 mL) and well water (2.07 log_10_ cfu/100 mL) (Table 1).

#### 3.1.6. Yeasts and Moulds

**Figure 5:**
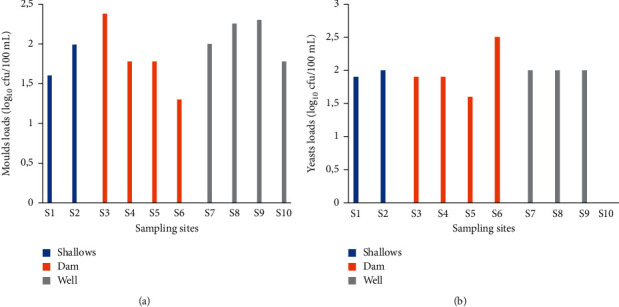
Moulds (a) and yeasts (b) counts in irrigation water.

Figures 5(a) and 5(b) show the yeast and mould counts from the sampling sites. Mould loads ranged between 1.3 and 2.38 log10 cfu/100 mL were obtained in irrigation water. These loads were recorded in sites 6 and 3, respectively. The yeast loads obtained ranged from 0 to 2.5 log_10_ cfu/100 mL. The maximum load of 2.5 log_10_ cfu/100 mL was obtained in water of site 6. An identical load of 2 log_10_ cfu/100 mL was obtained in water of sites 7, 8, and 9.

The average loads of moulds and yeasts are given in Table 1. The average mould loads were 1.8 log_10_ cfu/100 mL (shallows water), 1.81 log_10_ cfu/100 mL (dam water), and 2.08 log_10_ cfu/100 mL (well water). Water from wells had the highest average load. The average yeast loads were 1.95, 1.98, and 1.5 2.5 log10 cfu/100 mL for shallow, dam, and well water, respectively. The highest average load was obtained in water of shallow.

The different mould strains isolated from the irrigation water are given in Table 2. From the macroscopic and microscopic characteristics, four different mould strains were identified in irrigation water. These strains belong to the genus *Aspergillus* (Table 2).

#### 3.1.7. Classification of Sampling Areas

**Figure 6:**
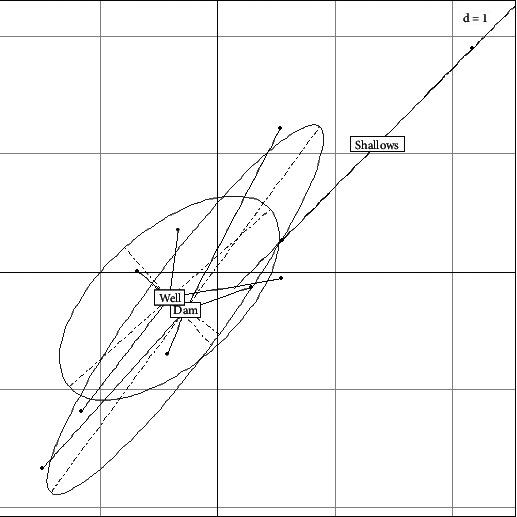
Classification of sampling locations according to the set of microorganisms.

Water from sites located in the shallows was overall the most contaminated (Figure 6). The circle of all well water loads and the circle of dam water loads overlap. Thus, there is no significant difference between the overall loadings of well water and dam water.

### 3.2. Physicochemical Analysis

#### 3.2.1. pH

**Figure 7:**
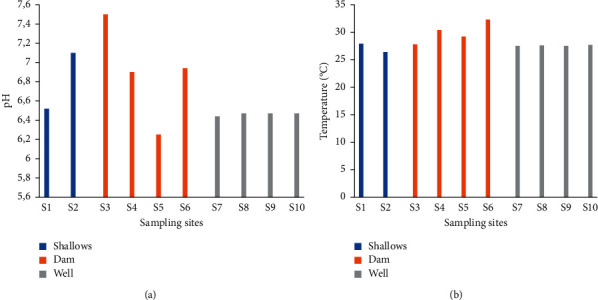
pH (a) and temperature (b) of irrigation water.

The pH of the irrigation water obtained ranged between 6.25 and 7.5 (Figure 7(a)). The pH of water at sites 8, 9, and 10 was identical (6.47). Except for the sites 2 and 3, water at the other sites was acidic. The average pH values obtained were 6.9, 6.81, and 6.46 for dam, shallow, and well water, respectively (Table 3). Overall, irrigation water was acidic.

#### 3.2.2. Temperature

The temperatures of irrigation water ranged from 26.4 to 32.3°C (Figure 7(b)). These temperatures were obtained at sites 2 and 6. The temperatures of the irrigation water at sites 3, 4, 5, and 6 were high compared to the water temperatures at the other sites. The average temperatures recorded were 27.1°C (shallow water), 27.5°C (well water), and 29.9°C (dam water) (Table 3).

#### 3.2.3. Electrical Conductivity

**Figure 8:**
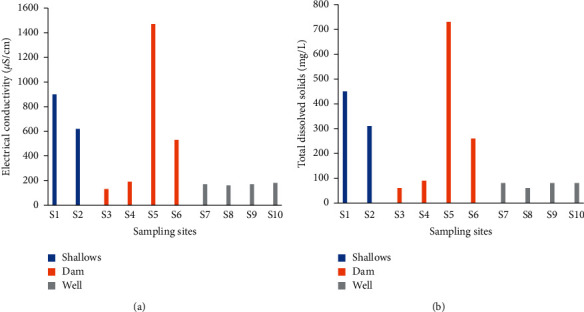
Electrical conductivity (a) and total dissolved solids (b) of irrigation water.

The minimum (130 *μ*S/cm) and maximum (1470 *μ*S/cm) conductivities of the irrigation water were recorded at sites 3 and 5, respectively (Figure 8(a)). In sites 7, 8, 9, and 10 (wells),the variation in conductivity was small compared to the sites in the other two. On average, conductivities of 170 *μ*S/cm, 580 *μ*S/cm, and 760 *μ*S/cm were obtained in well, dam, and shallow irrigation water, respectively (Table 3).

#### 3.2.4. Total Dissolved Solids

Total dissolved solids (TDS) concentrations ranging from 60 to 730 mg/L were obtained (Figure 8(b)). The site-specific characteristics of the TDS concentrations of irrigation water were identical to those of the conductivity. The average TDS concentrations were 70 mg/L (well water), 280 mg/L (dam water), and 380 mg/L (shallow water) (Table 3).

#### 3.2.5. Nitrate Concentration

**Figure 9:**
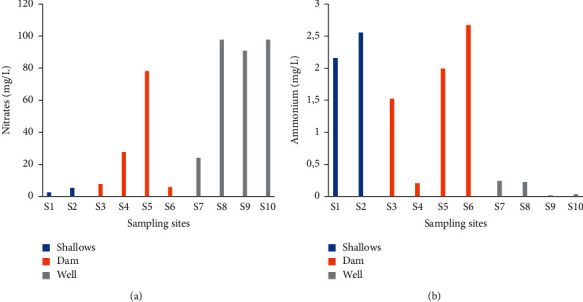
Nitrate (a) and ammonium (b) concentrations in irrigation water.

Nitrate concentrations ranging from 2.45 (site 1) to 97.75 mg/L were obtained in irrigation water (Figure 9(a)). Nitrate concentrations in well water were the highest. The lowest nitrate concentrations were obtained in shallow irrigation water (sites 1 and 2). The average nitrate concentrations of shallow, dam, and well water were 3.85 mg/L, 29.8 mg/L, and 77.59 mg/L, respectively (Table 3).

#### 3.2.6. Ammonium Concentration

Ammonium concentrations ranging from 0.016 to 2.67 mg/L were obtained in irrigation water (Figure 9(b)). Ammonium concentrations were higher from dam and shallows sites. The lowest concentrations were recorded in well water. Average ammonium concentrations were 2.35 mg/L (shallows water), 1.6 mg/L (dam water), and 0.13 mg/L (well waters). Shallow water was the highest average concentration followed by dam water (Table 3).

### 3.3. Correlations between Physicochemical and Microbiological Parameters

**Figure 10:**
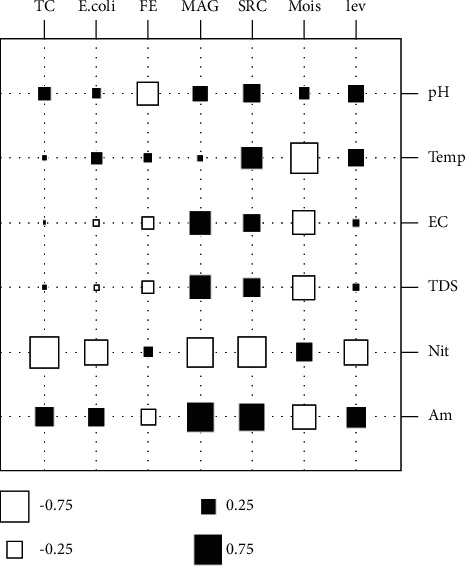
Correlation between physicochemical and microbiological parameters.

Aerobic mesophilic germs, total coliforms, sulphite-reducing clostridia, and yeasts are positively correlated to physical parameters (pH, temperature, conductivity, and TDS). This correlation is weak for total coliforms compared to the other three microorganisms. Fecal enterococci and moulds are each correlated to physical parameters, namely, temperature and pH. *E. coli* is positively correlated to pH and temperature. *E. coli*, mesophilic aerobic germs, total coliforms, sulphite-reducing clostridia, and yeasts are strongly correlated with ammonium. Fecal enterococci and moulds are positively correlated with nitrate (Figure 10).

## 4. Discussion

Water collected from all ten sites is contaminated by indicators of fecal contamination. This result corroborates that of Koffi-Nevry et al. [11]. These authors reported a 100% frequency of contamination by fecal contamination indicators (fecal coliforms, fecal enterococci, and *Clostridium perfringens*) in irrigation water in the periurban area of Abidjan. The mean loads of mesophilic aerobic germs obtained in this study are like those reported by Akinde et al. [15] in irrigation water in Nigeria. These authors obtained mesophilic aerobic germ loads between 5.10^4^ and 8.10^5^ cfu/100 mL in irrigation water. The group of aerobic mesophilic germs contains several germs (bacteria, yeasts, and moulds). All these germs are likely to contaminate irrigation water because most of these germs are ubiquitous. Regarding indicators of fecal contamination, total coliform loads obtained in this study are higher than those reported by Yapo et al. [16] in well water used for irrigation in Korhogo. The study conducted in Ghana on irrigation water by Abdallah and Mourad [17] showed higher *E. coli* loads than those obtained in the present study. Average loads of *E. coli* according to sampling locations are higher than the recommended value (2.3 log_10_ufc/100 mL). This result shows the very high level of microbial contamination in irrigation water. The loads of fecal enterococci and sulphite-reducing clostridia spores are lower and higher, respectively, than the study of Koffi-Nevry et al. [11]. Several sources could be responsible for fecal pollution of irrigation water. These are agricultural techniques and sewage from the city. Given the high cost of industrial fertilizers, market gardeners use large quantities of poultry and beef droppings as fertilizer for soil fertilization [11]. These practices are likely to contaminate irrigation water, especially those from wells. Indeed, these wells are shallow, uncovered, and located in market gardening sites. The wastewater is discharged into the environment, often without any treatment. This water, loaded with microorganisms, accumulates in receptacles such as dams and shallows, justifying the high contamination observed in these two places during this study. Unlike dam water, shallow water is stagnant. This characteristic could be the reason for the high contamination of shallows water compared to dam water. The fungal spores are present in air, soil, and plants and can contaminate irrigation water [18]. All the mould strains isolated belong to the genus *Aspergillus*. This result corroborates that of Akinde et al. [19] in the study on irrigation water and fresh vegetables in southwestern Nigeria. These authors also showed the ability of these strains to produce mycotoxins. The risk of contamination of vegetable products by irrigation water exists. Several studies have already shown this involvement of irrigation water in vegetable contamination [19–21].

The temperatures obtained in this study (26.4–32.3°C) are within the range of temperatures reported by Djegbe et al. [22] in irrigation water in Benin. The majority of the pH values measured for this study are acidic unlike those obtained by previous authors. According to Ahoussi et al. [23], acidity is one of the essential characteristics of water in Côte d'Ivoire. The electrical conductivity values recorded in well water used for irrigation are comparable to those Yapo et al. [16]. Ammonium concentrations are low (0.016–2.67 mg/L) compared to those obtained (0.022–5.98 mg/L) by Abdallah and Mourad [17]. Nitrate concentrations are well above those reported by Tano et al. [24]. According to Lagnika et al. [25], pollution sources for nitrogen salts include fertilizers, wastewater, animal waste, and septic tanks.

According to the coinertia results, mesophilic aerobic germs, sulphite-reducing clostridia, yeasts, and total coliforms have comparable environmental preferences (pH, temperature, conductivity, and TDS) in contrast to moulds and fecal enterococci. The strong correlation between pH and moulds reflects the acidophilic nature of most of these microorganisms. Mesophilic aerobic germs, sulphite-reducing clostridia, yeasts, total coliforms, and *E. coli* could come from the same sources of contamination as ammonium given the strong correlation between these parameters.

## 5. Conclusion

The irrigation water in the market gardening in Korhogo is polluted. Significant indicator loads (*E. coli*, fecal enterococci, and spores of sulphite-reducing clostridia) of fecal contamination were found in various irrigation water samples. Overall, water from shallows was the most contaminated. With the presence of these fecal bacteria, the irrigation water could harbor pathogenic germs. Thus, the risk of contamination of vegetables produced in market gardening sites by irrigation water exists. In addition, moulds of the genus *Aspergillus* have been identified in irrigation water. Some species of this genus can produce heat-resistant mycotoxins. The consumption of raw vegetable products without any disinfection could create a public health problem. Preventive measures must be taken to avoid cases of collective food poisoning. Raw market garden products must be properly disinfected before consumption. Simple and less expensive irrigation water treatments should be offered to market gardeners.

## Figures and Tables

**Table 1:** Average loads of microorganisms according to sampling locations.

Location	Microorganism loads (log_10_ufc/100 mL)						
	TC	*E. coli*	FE	MAG	SRC	Moulds	Yeasts
Shallows	4.35 ± 0.45	3.31 ± 0.71	2.89 ± 0.04	5.98 ± 0.22	3.63 ± 0.68	1.8 ± 0.27	1.95 ± 0.06
Dam	3.7 ± 0.36	2.44 ± 0.75	2.44 ± 0.71	5.34 ± 0.07	3.61 ± 0.61	1.81 ± 0.44	1.98 ± 0.37
Well	3.64 ± 0.28	2.5 ± 0.41	2.9 ± 0.09	4.95 ± 0.29	2.07 ± 1.4	2.08 ± 0.24	1.5 ± 1

TC, total coliforms; FE, fecal enterococci; MAG, mesophilic aerobic germs; SRC, sulphite-reducing clostridia.

**Table 2:** Macroscopic and microscopic aspect of mould strains.

Macroscopic aspect		Microscopic aspect	Strains
Obverse	Reverse		
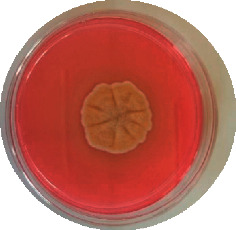	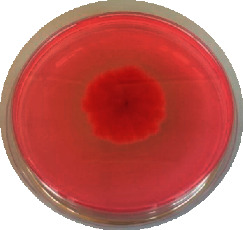	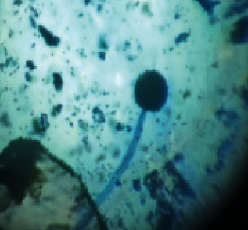	*Aspergillus* sp1
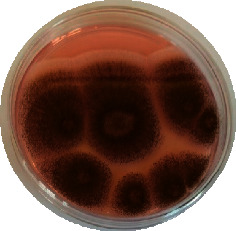	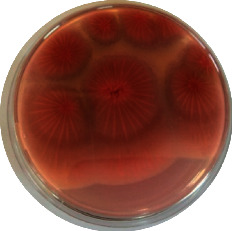	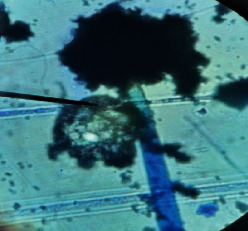	*Aspergillus* sp2
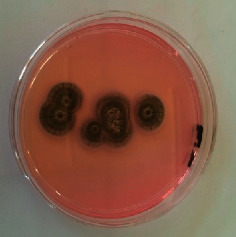	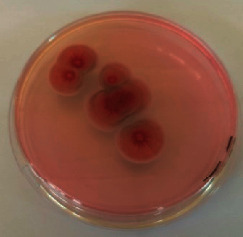	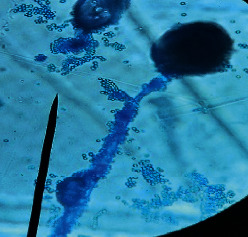	*Aspergillus* sp3
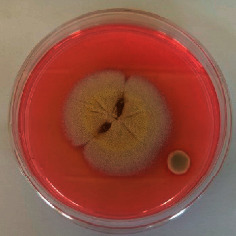	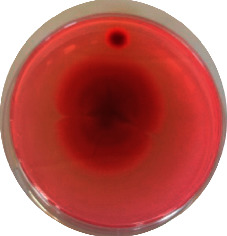	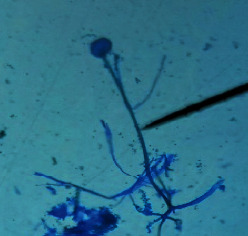	*Aspergillus* sp4

**Table 3:** Averages of physicochemical parameters according to sampling locations.

Sites	Physicochemical parameters					
	pH	Temperature (°C)	Electrical conductivity (*μ*S/cm)	STD (mg/L)	Nitrates (mg/L)	Ammonium (mg/L)
Shallows	6.81 ± 0.41	27.15 ± 1.06	760 ± 19	380 ± 90	3.85 ± 1.97	2.35 ± 0.28
Dam	6.9 ± 0.51	29.92 ± 1.9	580 ± 61	280 ± 20	29.8 ± 23	1.6 ± 1.04
Well	6.46 ± 0.01	27.57 ± 0.09	170 ± 0.8	70 ± 10	77.59 ± 35.82	0.13 ± 0.12

## Data Availability

The data used to support the findings of this study are included within the article.
